# Preliminary Study of a Millimeter Wave FMCW InSAR for UAS Indoor Navigation

**DOI:** 10.3390/s150202309

**Published:** 2015-01-22

**Authors:** Antonio F. Scannapieco, Alfredo Renga, Antonio Moccia

**Affiliations:** Department of Industrial Engineering, University of Naples Federico II, Piazzale Tecchio 80, Naples 80125, Italy; E-Mails: alfredo.renga@unina.it (A.R.); antonio.moccia@unina.it (A.M.)

**Keywords:** Synthetic Aperture Radar (SAR), interferometry, unmanned aerial systems (UAS), indoor, navigation, frequency-modulated continuous wave (FMCW), millimeter wave

## Abstract

Small autonomous unmanned aerial systems (UAS) could be used for indoor inspection in emergency missions, such as damage assessment or the search for survivors in dangerous environments, e.g., power plants, underground railways, mines and industrial warehouses. Two basic functions are required to carry out these tasks, that is autonomous GPS-denied navigation with obstacle detection and high-resolution 3D mapping with moving target detection. State-of-the-art sensors for UAS are very sensitive to environmental conditions and often fail in the case of poor visibility caused by dust, fog, smoke, flames or other factors that are met as nominal mission scenarios when operating indoors. This paper is a preliminary study concerning an innovative radar sensor based on the interferometric Synthetic Aperture Radar (SAR) principle, which has the potential to satisfy stringent requirements set by indoor autonomous operation. An architectural solution based on a frequency-modulated continuous wave (FMCW) scheme is proposed after a detailed analysis of existing compact and lightweight SAR. A preliminary system design is obtained, and the main imaging peculiarities of the novel sensor are discussed, demonstrating that high-resolution, high-quality observation of an assigned control volume can be achieved.

## 1. Introduction

Unmanned aerial systems (UAS) are commonly defined as medium-small scale uninhabited aerial vehicles able to attain stable flight operation thanks to a control system that can be programmed to follow a certain flight path or can be remotely controlled from a ground station. Today, UAS are moving toward autonomous sense and detect functions [1,2] and are performing missions with increasing levels of autonomy and complexity, such as repetitive reconnaissance and surveillance, whereby human presence onboard is undesirable or inadvisable. Outdoor flying unmanned vehicles have received a considerable amount of research and industrial attention over the years. Although limitations exist concerning UAS inclusion in air space, today, complete systems are available for military and civilian applications [3,4].

On the contrary, there is still much to be done in the area of indoor or urban autonomous operation, both for vehicle navigation and for monitoring or exploration. The application to unknown building interiors and very cluttered urban or natural environments is one of the most demanding issues envisioned for UAS, since it requires the real-time capability: (i) to detect and identify very different objects, such as buildings, walls, caves, infrastructures or underground facilities, in problematic and unpredictable illumination conditions; (ii) to navigate through complex-shaped passageways, even avoiding non-stationary obstacles; and (iii) to gather and relay information. Use of very compact sized and extreme lightweight small UAS or micro aerial vehicles (MAV), different from outdoor applications, represents an additional strong constraint when indoor flight operations must be performed. Target mission scenarios include high risk indoor inspection, e.g., nuclear power plant failure and leakage or tunnel roof collapse in mine, but also the search for survivors in cluttered dense urban environment or indoors, such as underground railways or industrial warehouses. Pipeline inspection and nuclear, biological or chemical (NBC) emergency reconnaissance represent additional dangerous applications that could take full advantage of small UAS and MAV operations. Completely different scenarios, but similar capabilities, are required in planetary exploration. Specifically, in past decades, rovers have emerged as one of the most important tools for planetary exploration. Important drawbacks of rover systems deal with the limited coverage they can achieve and uncertainty in terrain. For planetary and planet-like bodies, when a significant atmosphere is present, the above limitations can be overcome by aerial vehicles. In addition to Earth, several planets, such as Venus, Mars, Jupiter, Saturn, Uranus and Neptune, but also the Saturn moon, Titan, are endowed with an adequate atmosphere. Aerial vehicles proposed and investigated for planetary exploration include [5–7] airplanes and gliders, helicopters, balloons and airships. The most investigated solutions are based on lighter-than-atmosphere robotic airships combining the long-term airborne capability of balloons with the maneuverability of airplanes or helicopters.

The introduced applications involve flight operation in GPS-denied and substantially unknown environments with a potentially large communication latency (planetary explorations) or extended communication blackout periods (indoor emergencies). The accomplishment of two basic functions is required to carry out these tasks: fully autonomous navigation with obstacle detection/avoidance capability and high resolution 3D mapping and monitoring of the target area, including moving target detection. Unless the small UAS is provided with hovering capability, autonomous navigation presents clearly the most stringent time requirements. Regarding obstacle avoidance, in theory, accurate geometric models of the operational environment combined with thematic information and the description of all of the present objects could reduce the need for continuous and real-time sensing. However, those data are often neither updated nor available at the required spatial resolution and accuracy. Furthermore, unexpected obstacles, for instance consequent to an accident that requires to investigation, can appear anytime and anywhere; hence, real-time mapping capabilities are required, too.

The set of data needed to perform these tasks cannot be provided by sensors that are potentially adequate under conventional operating conditions, such as laser scanners and optical cameras, owing to their physical size, weight, strong dependence on illumination conditions and possible poor visibility caused by environmental factors. Conversely, radar sensors are able to operate in any illumination condition, and microwave carrier frequencies allow for coherent signal detection to be performed, thus resulting in significantly increased sensitivity and instant access to range information. In addition, high-resolution 3D mapping can be provided by combining the Synthetic Aperture Radar (SAR) technique with radar interferometry [8,9]. This also makes velocity information available via Doppler processing, which is a valuable feature for sensors operating onboard moving platforms. Finally, millimeter wave radar technology has been receiving increasing interest for application in small UAS [10,11] thanks to the limited size and power requirements and the capability to penetrate smoke and fire [12,13].

The objective of this work is to assess the main features, possible architectural schemes and technical solutions and to carry out a preliminary design of a very innovative radar sensor for novel autonomous operations onboard small UAS. Table 1 summarizes the key driving issues in the preliminary design that will be presented in the paper. First of all, it should be noted that for matching with the considered operational scenarios, the sensor must be compact, lightweight and characterized by low power consumption. In addition, it has to guarantee very high 3D resolution and accuracy, as well as the capability to perform real-time onboard processing in order to support autonomous navigation, exploration and mapping in completely unknown and unstructured environments.

## 2. System Architecture

### 2.1. State-of-the-Art Analysis

In the last decade, several compact and lightweight SARs have been developed and tested for different purposes and applications. Table 2 lists the most relevant systems together with their main features, as available today in the open literature. All of them are devoted to outdoor operations, such as surveillance and remote sensing applications, and work in side-looking mode with limited pointing capability. Vision-based navigation through those radar sensors has not been implemented yet. None of these systems satisfies all of the constraints of Table 1. Real-time onboard operation is rarely enabled; resolutions can be insufficient; and in most cases, the mass and power requirements exceed small platform availability. Nonetheless, a few interesting features can be highlighted. MiniSAR by Sandia National Labs [14] and Miniaturized SAR (MISAR) by European Aeronautic Defence and Space Company N.V. (EADS) [11]; both include a double gimbal structure, which allows mechanical steering of the antenna to be achieved, thus making SAR interferometry along multiple directions possible. In both cases, two separate antennas, one for transmission and one for reception, are accommodated to implement a frequency-modulated continuous wave (FMCW) scheme. More than half of the listed sensors exploit this architectural scheme, even though not possessing a gimbal structure. Finally, it is important to remark that AiR-Based REmote Sensing (ARBRES) X-Band SAR [15] and MetaSensing X-Band SAR [16] make use of three antennas, namely two receiving and one transmitting for performing FMCW single-pass interferometry.

In the following subsections, a critical analysis of some key design solutions is presented, and then, an adequate innovative architecture is proposed.

### 2.2. Why FMCW SAR

First of all, it is necessary to point out the advantages connected to the use of FMCW SAR. FMCW radar transmits a frequency-modulated signal, which is usual in SAR, but in a continuous wave, differently from most realizations. The received echo, which is delayed by round trip delay τ associated with target-range distance, is mixed with the transmitted signal [17]. For a linear frequency modulation, the output of the mixing process, namely the beat signal, has two Fourier components at different frequencies. The first component is a signal centered at a constant frequency lower than the carrier frequency [18]. The second component is a residual signal centered approximately at twice the carrier frequency, which has less energy with respect to the former component [17] and is filtered out. The process involving both the mixing of transmitted and received signals and the low-pass filtering of a beat signal is also called deramp-on-receive.

The aforementioned low, constant frequency in the beat signal, which is computed by differentiating the phase term of the beat signal with respect to time, is labeled as the beat frequency. The beat frequency holds strong relevance in FMCW radar, as it is directly proportional to the target range by the ratio between the propagation velocity and the bandwidth of the transmitted signal, thus allowing the system to compute the range by measuring the beat frequency. The theoretical value for the range resolution is [17]:

(1) $$dr=\frac{c}{2B}$$

where *c* is light velocity and *B* is the transmitted bandwidth. Actually, Equation (1) is equivalent to the conventional pulsed radar theoretical range resolution [8,36]. However, it is important to remark that the FMCW range compressed signal is obtained in the frequency domain rather than in the time domain.

The FMCW scheme guarantees decisive advantages with respect to conventional pulsed SAR, especially when compact systems have to be realized. Continuous transmission, *i.e.*, a unity duty cycle *η* = 1, involves less transmitted peak power, which makes significant simplifications in the power generation and conditioning unit along with a strong reduction in power requirements with respect to pulsed systems possible. In addition, deramp-on-receive relies on the sampling of the beat signal bandwidth *B_B_* instead of the whole transmitted bandwidth *B*. This means that even the GHz bandwidth can be easily handled by a MHz sampling frequency *f_S_*, because *B_B_*≪*B*, thus allowing simpler and cheaper hardware equipment.

The FMCW's peculiar features in comparison to traditional pulsed technology are consequent to the motion during continuous transmission. A better understanding of motion effects on the signal is given by [37] in which the following equation is reported for the beat signal in the two-dimensional spatial frequency domain:

(2) $$S_{B}\left(K_{r},K_{x}\right)=\exp(jK_{x}vt)\exp\left(jR_{0}\sqrt{K_{r}^{2}-K_{x}^{2}}\right)$$

where *K_r_* and *K_x_* are the spatial frequencies in the range and azimuth directions, respectively, *υ* is the platform velocity, *R*_0_ is the distance of the closest approach and *t* is the time referring to the signal transmission/reception at velocity *c*. The second exponential in Equation (2) coincides with the beat signal of conventional pulsed SAR in the two-dimensional spatial frequency domain, whereas the first is a space invariant term that takes into account the motion during transmission. This term becomes equal to one in conventional SAR, because of the start-stop approximation, which assumes that the radar is stationary during the pulse transmission-reception, because *v* ≫ *c*. Start-stop approximation is traditionally exploited to explain raw SAR image formation [8]. As a direct consequence of Equation (2), in general, conventional algorithms for SAR image formation would result in FMCW SAR image degradation. More complex reference functions have to be adopted in these cases [38].

**Figure 1.**
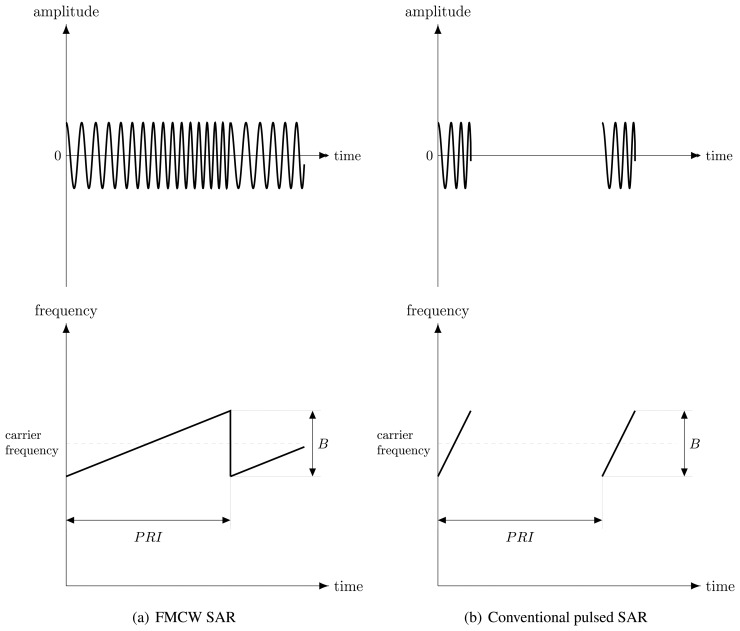
Comparison between pulse repetition interval (PRI) (**a**) in FMCW SAR and (**b**) in conventional pulsed SAR. The lots are not to scale for clarity.

However, specific conditions exist in which start-stop approximation can be considered valid for FMCW SAR, too. Even though continuous transmission is used, it is possible to define the concept of the pulse repetition interval (PRI) for FMCW radar as the sweep duration, *i.e.*, the time the transmitted frequency takes to shift from the minimum to the maximum frequency, or equivalently, the time between the start of two consecutive sweeps. It is clear that the last definition leads to almost a similar PRI meaning as for pulsed SAR, although it refers to sweep instead of chirp (see Figure 1). Based on the introduced PRI, the pulse repetition frequency can be defined as the reciprocal of the PRI.

The Nyquist sampling theorem requires PRI to be small enough in order to properly sample the azimuth Doppler history. In detail, provided that the sampling requirements are satisfied [38], each sweep represents a sample of the Doppler history in the same way as a pulse of conventional SAR. Hence, both fast time *t* and slow time *t_N_* (*i.e.*, referring to radar motion at velocity *v)* can be introduced for FMCW SAR, too. On the other hand, a longer sweep duration would produce several samples in the azimuth Doppler history within each sweep, thus making start-stop approximation less acceptable. The remainder of this paper focuses on the case in which start-stop approximation is valid [16,38].

As in conventional SAR, the FMCW SAR target response exhibits a Doppler bandwidth, *B_D_*, generated by the variation of the observation angle and, therefore, by the variation of the radial velocity:

(3) $$B_{D}=2\frac{v}{\lambda }\left[\sin\left(\theta_{sq}+\frac{\theta_{\text{az}}}{2}\right)-\sin\left(\theta_{sq}-\frac{\theta_{\text{az}}}{2}\right)\right]$$

where *λ* is the carrier wavelength, *θ_sq_* is the squint angle and *θ_az_* is the beamwidth in the azimuth direction. Hence, provided that proper motion compensation algorithms are exploited [17,38], the theoretical FMCW SAR azimuth resolution is:

(4) $$da=\frac{v}{B_{D}}=\frac{l_{\text{az}}}{2}$$

where *l*_az_ is the antenna length. Equation (4) is exactly the same equation that holds for conventional pulsed SAR.

As expected, the result of range and azimuth compression is a bi-dimensional sinc function multiplied by two complex exponentials, the former depending on both the minimum platform to target distance and a reference distance *R*_ref_ used for the processing [39], the latter depending only on the reference distance and system parameters. Namely:

(5) $$s(f_{R},t_{N})=\text{sinc}\left[\pi \left(f_{R}+\frac{R_{0}-R_{\text{ref}}}{\text{cPRI}}2B\right)\left(\text{PRI}-2\frac{R_{0}}{c}-\frac{v^{2}t_{N}^{2}}{cR_{0}}\right)\right]\cdot \text{sinc}\left[B_{D}\left(t_{N}-\frac{x_{0}}{v}\right)\right]B_{D}\exp\left[-j\frac{4\pi }{\lambda }(R_{0}-R_{\text{ref}})\right]\exp\left(-j\pi \frac{B}{\text{PRI}}\tau_{\text{ref}}^{2}\right)$$

where *f_R_* is the range frequency, *x*_0_is the position of the target along the azimuth direction with respect to the center scene and *τ*_ref_ is the time delay of the echo at reference range *R_ref_*, which corresponds to the range from the center scene. The first exponential resembles the exponential term of the pulsed SAR 2D-focused signal and again can be exploited to perform interferometry (see Section 2.3). Moreover, it has to be noted that the signal of Equation (5), unlike the pulsed SAR 2D-focused signal, is better described in the range-time domain, as range frequency *f_R_* is directly proportional to the range in FMCW SAR. Finally, the amplitude of the resulting signal depends on the Doppler bandwidth.

The implementation advantages of FMCW SAR must be weighed against some drawbacks that this scheme exhibits. In general, data processing is more complex with respect to pulsed SAR, because deramp-on-receive produces an unwanted phase term, called the residual video phase (RVP), which must be removed. In addition, moving targets can introduce ambiguities in range measurement. Indeed, owing to longer observation time compared to a conventional system, targets can move through several resolution cells within a sweep [38], causing the Doppler effect not to be negligible. Several solutions have been proposed to correctly determine the range, even in the presence of moving targets, including triangular frequency modulation [17,18] to determine the range and Doppler information within a single time interval. Non-linearities in transmitted and received signals cause an additional erroneous phase term in the beat signal, therefore leading to deteriorated range resolution [38]. Typical algorithms for non-linearity correction work under the assumption that non-linearity effects depend linearly on time delay, which is true for small distances. This is the case of indoor applications. The assumption falls for long range observations and causes the computational load to increase. Hardware and software solutions are known in the literature [17,38], such as voltage-controlled oscillator (VCO) and direct digital synthesizer (DDS), or approaches based on approximations of non-linearity. Finally, the simultaneous signal transmission and reception generate signal leakage in the reception chain. Specifically, due to the extremely high transmitted-to-received power ratio, saturation or damage of equipment can occur if even a small leakage of transmitted power is present [18]. Good isolation is therefore required, and typically, separated transmitting and receiving antennas in both bistatic and quasi-monostatic configurations are exploited. Considering that relatively assessed solutions are today available to deal with the discussed drawbacks and taking into account its advantages for the considered applications, the FMCW SAR scheme is selected herein as a base for the system architecture.

### 2.3. Why SAR Interferometry

SAR interferometry is a technique that exploits phase information, obtained from two or more SAR images, in order to compute target height and position in a three-dimensional environment. It can be considered a well-assessed technology for conventional pulsed SAR [8,9]. As regards FMCW SAR, the 2D-focused SAR signal (see Equation (5)) shows that the phase of the azimuth sinc samples target range as the multiple of the wavelength and can therefore be utilized to perform interferometry. It has to be noted that it is necessary to remove the additional contribution to the phase given by the reference range distance, which is typically the distance to the center of the scene illuminated by the beamwidth, and therefore, it can be different in the two images to be correlated. SAR interferometry has been successfully tested on data collected by FMCW SAR [16], and it is considered a key asset towards the operational scenario considered in this work.

### 2.4. Selected Scheme

**Figure 2.**
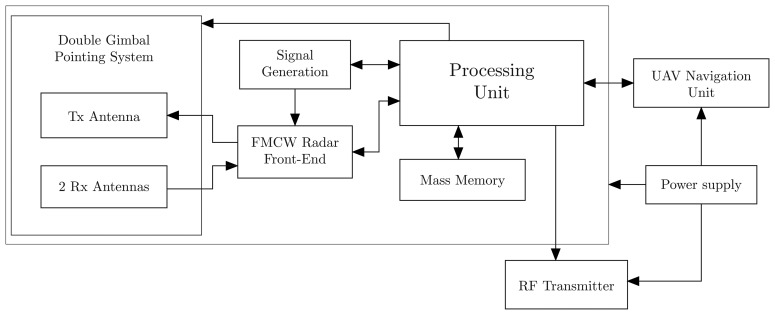
System architecture.

Based on the state-of-the-art analysis, a system architecture that is potentially able to satisfy all requirements listed in Table 1 is shown in Figure 2. The selected scheme is an interferometric FMCW SAR, equipped with three antennas, one transmitting and two receiving, mounted on a double gimbal structure. Among various factors, interferometric measurement resolution and accuracy are strongly dependent on antenna separation knowledge and control. Furthermore, the proposed system is compact and operates on a single platform, *i.e.*, the two antennas could be rigidly connected and simultaneously pointed to specific targets by adequately rotating a double gimbal to change the baseline (*i.e.*, antenna separation with respect to the target). Hence, it is expected to achieve adequate performance. It is worth noting that: (i) although electronic antenna steering would be favorable for fast and accurate sweeping of all hemispherical field-of-views, the creation of adequate baseline components to extract phase measurements is based on antenna mechanical re-orientation; consequently, the design and development of a double gimbal has been considered to make easier realization of both the antenna and electronics; (ii) depending on the platform selected for the mission, for instance a quadrotor, antenna mechanical re-orientation can be achieved by either rotation of the platform itself or the combined action of the platform and double gimbal.

In addition, an autonomous processing unit (PU), committed to real-time onboard data processing, is included in the scheme. Radar data are stored onboard in a mass memory unit. These data are exploited by the PU to directly command the double gimbal pointing system. The PU also sends information to the UAS navigation unit via a direct interface data link. Communication from the navigation unit to the PU is also necessary to support image processing and data extraction. Finally, the PU interfaces with the radio frequency transmitter to send stored data to the ground station via a wireless data link, when available.

## 3. Preliminary System Design

### 3.1. Preliminary Design Process

**Figure 3.**
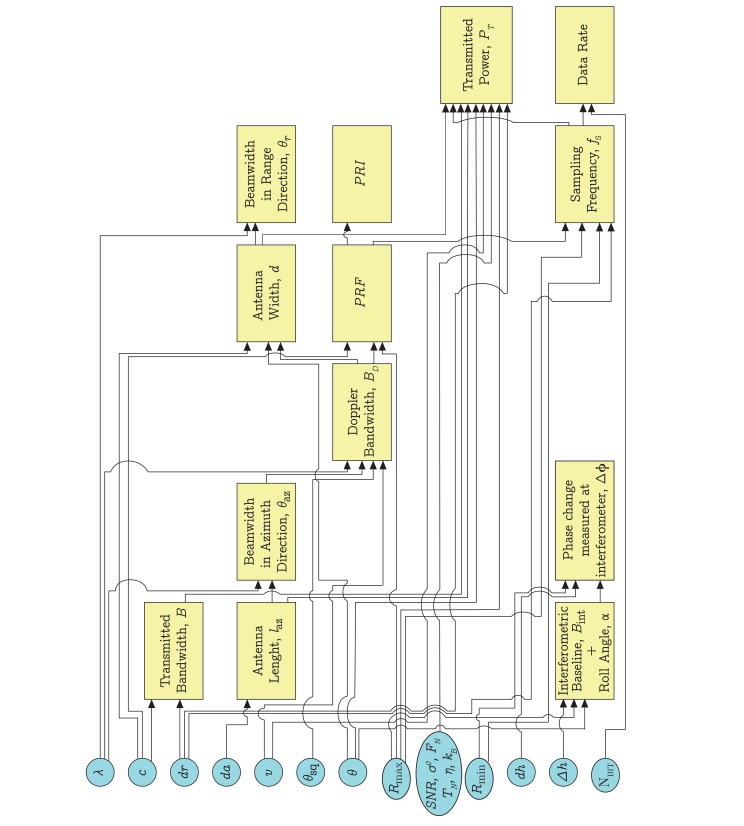
Design process guidelines: Block diagram.

The design process is outlined in Figure 3: circles represent input parameters, which have been chosen according to the system requirements (Table 1), the system architecture (Figure 2) and the application, whereas boxes return the sought values. The input parameters of the design process are chosen first. Table 3 lists the input parameters that vary within a minimum and maximum value, whereas Table 4 lists the ones that assume a constant value in the implemented design process.

The resolution requirements in range, azimuth and height directions are chosen according to the expected performance, whereas boundaries on platform velocity and maximum and minimum range distances depend on the application. In our case, it is the dynamics of the small UAS flying in an indoor environment performing loitering maneuvers. In addition, a typical value for an indoor differential radar cross-section has been considered. The following sub-sections report a brief explanation of peculiar blocks, specific for the FMCW SAR design. An example of the overall system characteristics is finally derived, accordingly.

### 3.2. Ambiguities and Antenna Width

**Figure 4.**
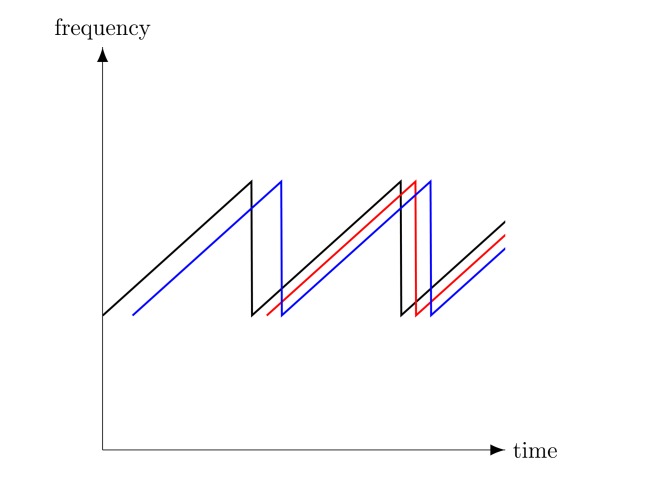
FMCW ambiguity in range: The first sweep reflection from the furthest target (red line) is between the transmitted signal (black line) and the second sweep reflection from the closest target (blue line), so that the furthest target is imaged closer.

Range ambiguity for a FMCW radar may occur owing to the continuous transmission of a frequency modulated signal when an echo from a target arrives at receiver after the end of the sweep that generated it. As a result, the received signal will be mixed with a different sweep and will result in the target being closer than in reality (see Figure 4). The unambiguous range is therefore equal to the round-trip distance covered by the wave in a single sweep, namely:

(6) $$R_{u}=\frac{\text{cPRI}}{2}$$

Therefore, under the hypothesis that the whole swath width is less than the unambiguous range, the following inequalities shall be satisfied to avoid echo ambiguities and bandwidth undersampling:

(7) $$\frac{c}{2(R_{\text{FR}}-R_{\text{NR}})}>\text{PRF}>2B_{D}$$

where the subscripts FR and NR refer to far- and near-range, respectively. The difference *R*_FR_ − R_NR_ depends on the antenna aperture, hence on the antenna width in elevation in an inverse proportion. Since the considered distances and the Doppler bandwidth are small, Equation (7) does not yield strict bounds on the antenna dimensions. Hence, the antenna width *d* can be quite small and may be chosen according to other requirements, e.g., the radar equation, heat dissipation and technological restrictions.

### 3.3. Transmitted Power

Transmitted power can be computed by the following formula derived in [40]:

(8) $$P_{T}=\frac{\text{SNR}{\left(4\pi \right)}^{3}R_{\max}^{4}k_{B}F_{N}T_{N}B_{N}}{G_{T}G_{R}\lambda^{2}\sigma^{0}dr_{\text{gr}}daN_{R}N_{A}}$$

which takes into account the range and azimuth compression gains, *N_R_* and *N_A_*, respectively. In Equation (8), the subscripts *T* and *R* refer to transmitting and receiving antenna gains *(G), B_N_* is the noise bandwidth and *dr_gr_* is the ground range resolution.

For rectangular antennas, the gain at the boresight is expressed in [41,42] as:

(9) $$G=k_{e}\frac{4\pi A}{\lambda^{2}}$$

where ke is an efficiency factor, typically equal to 0.65, and A the antenna area. Under the hypothesis of identical transmitting and receiving antennas and by expressing compression gains as in [43], Equation (8) becomes:

(10) $$P_{T}=\frac{\text{SNR}4\pi R_{\max}^{3}k_{B}F_{N}T_{N}B_{N}l_{\text{az}}v}{\eta {k_{e}}^{2}A^{2}\sigma^{0}dr_{\text{gr}}\text{daB}}$$

Concerning the transmitted power, it is important to point out that in FMCW SAR, noise bandwidth *B_N_* is equal to sampling frequency *f_S_* [44]. This is an additional advantage over conventional SAR, in which the noise bandwidth is equal to the transmitted one.

### 3.4. Interferometry

**Figure 5.**
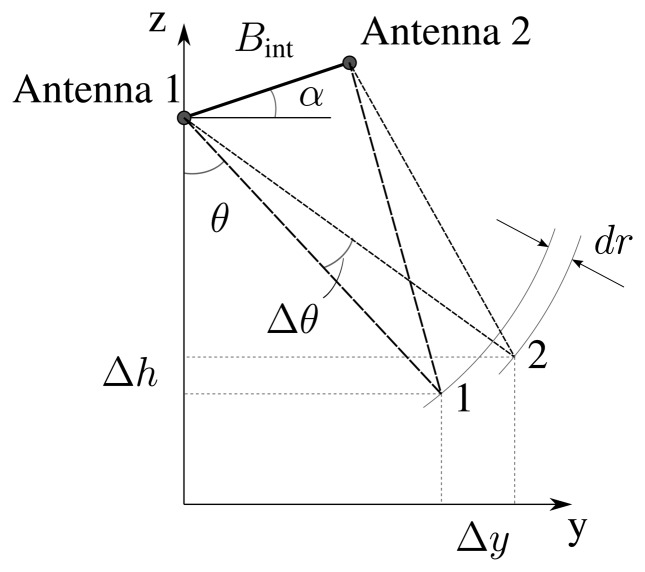
Interferometric observation geometry.

Plane wave approximation (pwa) is a typical assumption exploited to perform interferometry and to compute interferometric phase *φ*. With reference to the geometry depicted in Figure 5, this leads to:

(11) $$\phi_{1}=\frac{2\pi }{\lambda }(R_{2,1}-R_{1,1})\approx -\frac{2\pi }{\lambda }B_{\text{int}}\sin(\theta -\alpha )$$

where *B_int_* is the interferometric baseline defined as the modulus of the antenna separation vector and *α* is the baseline roll angle. In Equation (11) and following, *φ_i_* represents the interferometric phase of the *i*-th point and *R_j,i_* the distance between the *j*-th antenna and the *i*-th point. Therefore, the differential phase between two points in adjacent range cells, with separation in height Δ*h* and separation in slant range *dr* = *R*_1,2_ − *R*_1,1_, is:

(12) $$\Delta \Phi_{\text{pwa}}=\phi_{2}-\phi_{1}=-\frac{2\pi }{\lambda }B_{\text{int}}[\sin(\Delta \theta +\theta -\alpha )-\sin(\theta -\alpha )]$$

where:

(13) $$\Delta \theta =\cos^{-1}\left(\frac{R_{1,1}\cos\theta -\Delta h}{R_{1,1}+dr}\right)-\theta$$

is the variation in the off-nadir angle related to the difference in height.

For a close-range (cr) application, as is the aim of the present work, the plane wave approximation is not valid anymore. Hence, Equation (11) must be generalized as:

(14) $$\phi_{1}=\frac{2\pi }{\lambda }(R_{2,1}-R_{1,1})=\frac{2\pi }{\lambda }\left[\sqrt{R_{1,1}^{2}+B_{\text{int}}^{2}-R_{1,1}B_{\text{int}}\sin(\theta -\alpha )}-R_{1,1}\right]$$

thus leading to differential phase:

(15) $$\Delta \Phi_{\text{cr}}=\frac{2\pi }{\lambda }\left[\sqrt{R_{1,2}^{2}+B_{\text{int}}^{2}-R_{1,2}B_{\text{int}}\sin(\Delta \theta +\theta -\alpha )}-\sqrt{R_{1,1}^{2}+B_{\text{int}}^{2}-R_{1,1}B_{\text{int}}\sin(\theta -\alpha )}+R_{1,1}-R_{1,2}\right]$$

The percentage error resulting from the adoption of the plane wave approximation (12) in a close-range application can be calculated as:

(16) $${ɛ}_{\Delta \Phi }=\frac{\Delta \Phi_{\text{cr}}-\Delta \Phi_{\text{pwa}}}{2\pi }\times 100$$

**Figure 6.**
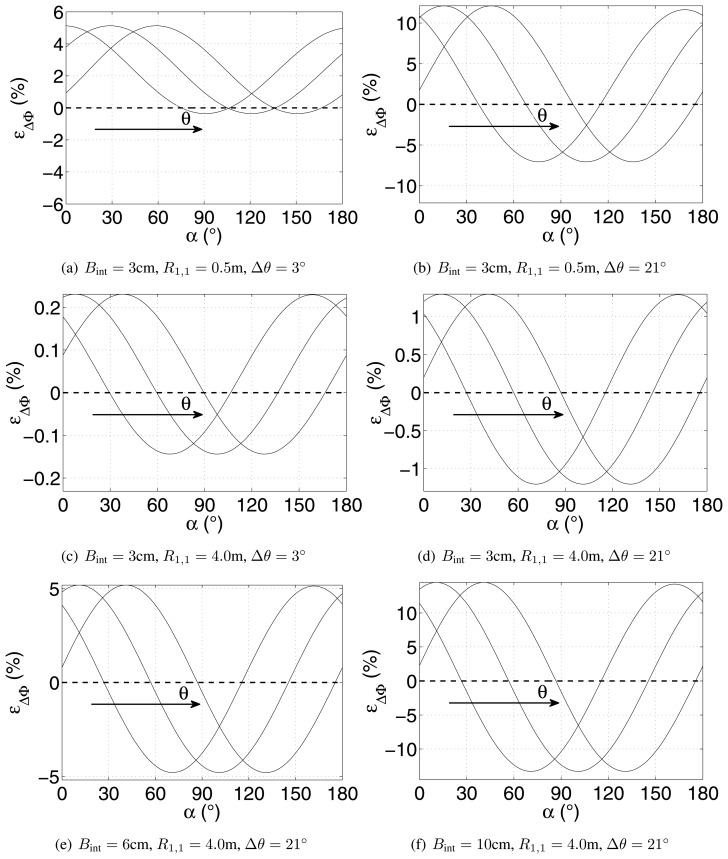
Percentage error between the true and approximated differential interferometric phases under various operating conditions (the three curves correspond to *θ* = 15°, *θ* = 45°, *θ* = 75°).

Figure 6 shows the percentage error function for various *θ*, Δ*θ*, *B*_int_ and *R*. The error increases for larger *B*_int_ and closer targets, as the line of sight of two antennas becomes less and less parallel. Finally, increasing the off-nadir angle *θ* causes a shift of the function towards larger *α*, although, obviously, the periodic behavior of the function is clear.

#### 3.4.1. Interferometric Baseline

**Figure 7.**
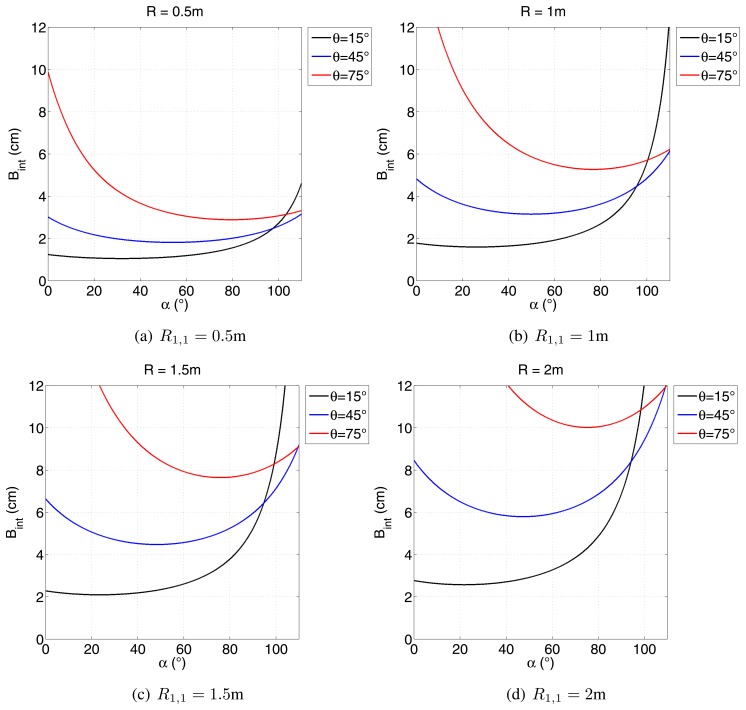
Critical baseline for various operating conditions. For each plot, *dr* = 10 cm and Δ*h* =10 cm have been considered.

**Figure 8.**
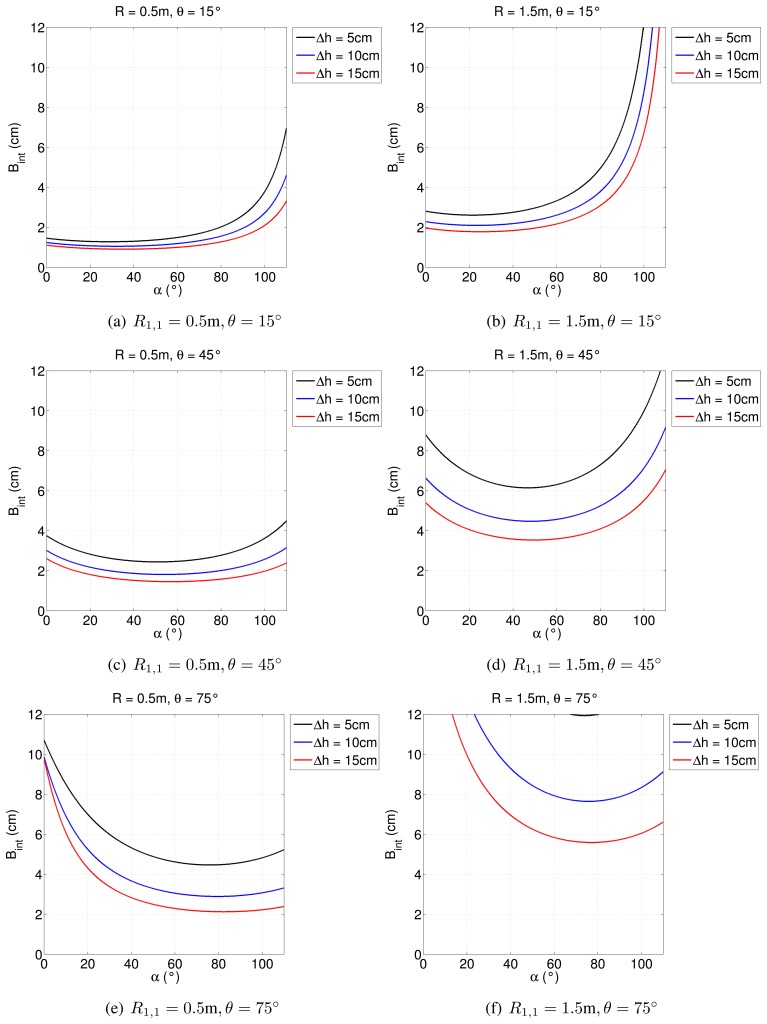
Effect of height variation on the critical baseline. For each plot, *dr* = 10 cm has been considered.

A new method to design the interferometric baseline for close-range applications is required. Equation (15) does not allow *B*_int_ to be obtained directly from the other parameters, so it is necessary to address an indirect solution. The one hereby proposed envisages exploiting the numerical representation of Equation (15), given a certain geometry, as a function of a range of values for both B_int_ and *α*. One of the requirements for the correct reconstruction of height variation is that the difference in phases between two adjacent pixels is no greater than 2π. Therefore, all of the couples:

(17) $$(B_{\text{int}},\alpha ):\Delta \Phi_{\text{cr}}(B_{\text{int}},\alpha )>2\pi$$

are discarded, whereas all of the other values could represent a good choice, depending on the application. The value of the maximum allowable interferometric baseline:

(18) $$B_{\text{int}}:\Delta \Phi_{\text{cr}}(B_{\text{int}})=2\pi$$

referred to as the critical baseline [9], is shown in Figures 7 and 8 for various operating conditions.

As expected, Figure 7 shows that when the range increases, the critical baseline increases, as well. This means that, depending on the size of the antennas, a minimum interferometric baseline is achievable, thus imposing a bound on the smallest distance at which it is possible to perform interferometry. Based on this consideration, minimum values for *R*_min_ listed in Table 3 have to be updated accordingly.

However, it has to be pointed out that this minimum distance is also strongly related to the height variation between points in adjacent range cells. Namely, if Δ*h* is smaller than expected, then interferometry can be performed at even a smaller range distance (see Figure 8).

### 3.5. System Parameters

In Section 3.1, input parameters, due to both requirements and the envisaged missions, for the design of an innovative FMCW SAR system have been shown (see Table 3). In the remainder of this section, attention will be paid to further assumptions, which have been made to achieve a combination of working parameters (see Table 5) by exploiting the design block diagram depicted in Figure 3 and by accounting for the radar and interferometry constraints previously discussed.

In order to propose an advanced configuration, the most stringent input values from Table 3 have been chosen for theoretical three-dimensional resolution. Furthermore, the mission profile contributed to the choice of both platform velocity *v*, small enough to move in unknown environments, and the expected difference in height Δ*h*, set equal to the height resolution. Finally, the off-nadir angle *θ*, which influences both transmitted power *P_T_* and interferometric performance, has been chosen to achieve an adequate baseline. It is worth noting that, being that the radar is designed to operate indoors, at close range, the transmitted power is much lower than the values of the existing, compact, lightweight systems listed in Table 2. Nonetheless, the parameters reported in Table 5 must be considered as nominal ones. From the practical point of view, the system must be able to collect useful data under extremely different operating conditions depending on the observation geometries, the synthetic aperture formation and the effective baseline. The next section will focus on these problems, which are critical for the proposed system.

## 4. Assessment of Three-Dimensional Mapping Capabilities

**Figure 9.**
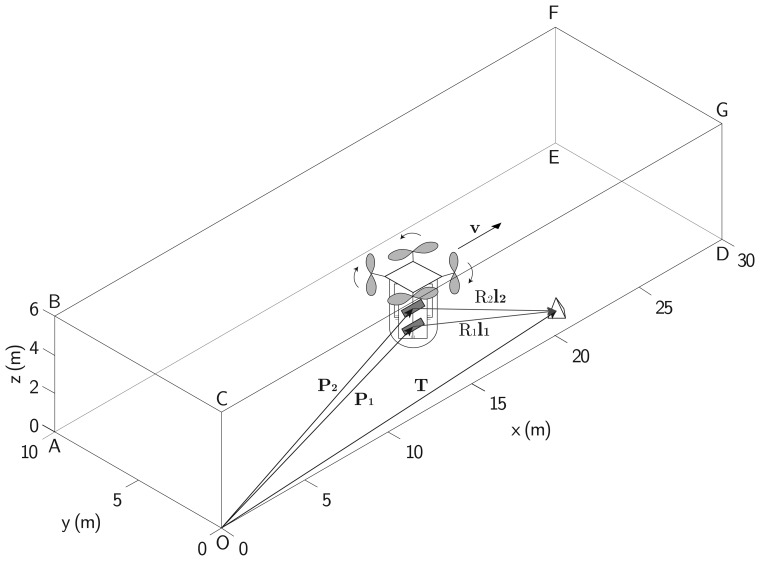
Platform and sensor moving in a simplified operational scenario. The platform and target position vectors, the line of sight unit vector, the velocity vector and the target distance to the antennas are depicted, too (not to scale, for clarity).

A typical operational scenario for the proposed system is well represented by a parallelepiped, whose dimensions are depicted in Figure 9. Specifically, concerning indoor exploration, this parallelepiped can represent an example of a warehouse in which the sensor is requested to operate. The same scenario is valid also for planetary exploration, where the parallelepiped can be conceived of as a relatively small control volume that encloses scatterers, which vary depending on the application.

The design values proposed in the previous section (see Table 5) allow both acceptable values of SNR for the whole range of distances to be obtained and the start-stop approximation to be exploited. Concerning geometric resolution, it is worth highlighting that a practically rectangular resolution element is achieved when a conventional side-looking monostatic SAR is considered. Specifically, this is possible because the azimuth or the along-track directions and ranges or the across-track direction are orthogonal and the sampling frequency and pulse repetition frequency (PRF) are tuned correspondingly, accounting for multilook processing, too [45]. On the contrary, the proposed system is designed to look in general along directions not perpendicular to the motion of the platform. As a result, image pixels no longer cover rectangular, but differently-skewed areas. Hence, in order to get satisfactory resolutions, it is of primary importance both to introduce a set of figures of merit to decide whether an image is acceptable or not and to evaluate the system performance in the control volume.

### 4.1. Geometric Model

The target position in three-dimensional space is determined by the intersection of three surfaces:

(19a) R=‖P−T‖

(19b) $$f_{D}=2\frac{\text{v}\cdot \text{l}}{\lambda }$$

(19c) $$\phi =\frac{2\pi }{\lambda }(R_{2}-R_{1})$$

namely the range sphere, Doppler cone and phase hyperboloid [9].

Given a Cartesian coordinate system, whose origin is in the vertex O and axes along the edges of parallelepiped OD, OA and OC in Figure 9, P and T represent the antenna and target positions in Equation (19), whereas **l** represents the line of sight vector. It is worth noting that, if plane wave approximation is valid [9], the phase hyperboloid Equation (19c) degenerates into a cone.

#### 4.1.1. Range Sphere-Doppler Cone Intersection

The gradient method can be exploited to assess the effects of pixel shape in the presence of the squint angle within the whole three-dimensional environment. The application of the gradient method requires the introduction of more general definitions of range and Doppler or azimuth directions as the direction of fast time gradient $\vec{\nabla t}$ and Doppler frequency gradient $\vec{\nabla f_{D}}$, respectively [46]. In addition, a further hypothesis of motion at constant velocity within the integration time is assumed. It is worth noting that the gradient method, traditionally applied considering terrain, can be extended to each wall in the case of indoor navigation to get a three-dimensional awareness.

The characteristics of range and Doppler isolines, caused by the intersection of both the range sphere and Doppler cone with walls, are analyzed herein. In detail, the unambiguous area is defined in the plane of each wall as the geometric locus that simultaneously satisfies the following three criteria:

- the angle Ωof intersection between the iso-range and iso-Doppler contour lines falls within the interval [Ω_min_, Ω_max_],
- the spatial resolutions computed along the range and Doppler directions are not lower than required in Table 1,
- the area of an illuminated pixel *(i.e.*, the area bounded by two adjacent iso-range and iso-Doppler lines) is smaller than a threshold *A*_pixel_ related to the required cell resolution.

Consequently, the ambiguous area is the complement of the unambiguous one. The aforementioned criteria physically mean that within the ambiguous area, the shape of the resolution cell does not allow the target position on the wall plane to be established with the desired accuracy, owing to the size of the resolution cell and the geometry of both the isolines and the pixel. Furthermore, it is worth noting that a phase value can be assigned to a point observable in both the range and Doppler domain, that is a point that lies in the unambiguous area, thus making interferometry possible.

The imaging performance is estimated considering the parameters listed in Table 6. The azimuth or Doppler resolution depends on the integration time or synthetic aperture duration. The integration time should be defined as the time span for which a given target is illuminated by the main lobe of the transmitting antenna and remains within the main lobe of the receiving one. For the considered system and environment, the integration time is a function of the distance and of the relative geometry between the sensor and the target. Hence, it varies from point to point within the control volume. However, since this actual integration time is, in general, not known, the performance analysis is addressed in this section by supposing a constant integration time. This means that the integration time must be interpreted herein as the time span used for SAR focusing, which is assumed constant for all of the imaged targets. The value for integration time reported in Table 6 is also compliant with the possible platform dynamics and antenna apertures assumed in the simulation. As a consequence, a range of distances at which the theoretical azimuth resolution (Equation (4)) can be achieved will exist. Farther points may suffer from worse resolution owing to the increasing distance between either two close iso-range or iso-Doppler curves, which results in a larger imaging pixel. Nonetheless, as shown in the following, the degraded pixel is still complaint with the minimum required resolution and pixel area threshold (Table 6) over sufficiently large areas within the test environment.

**Figure 10.**
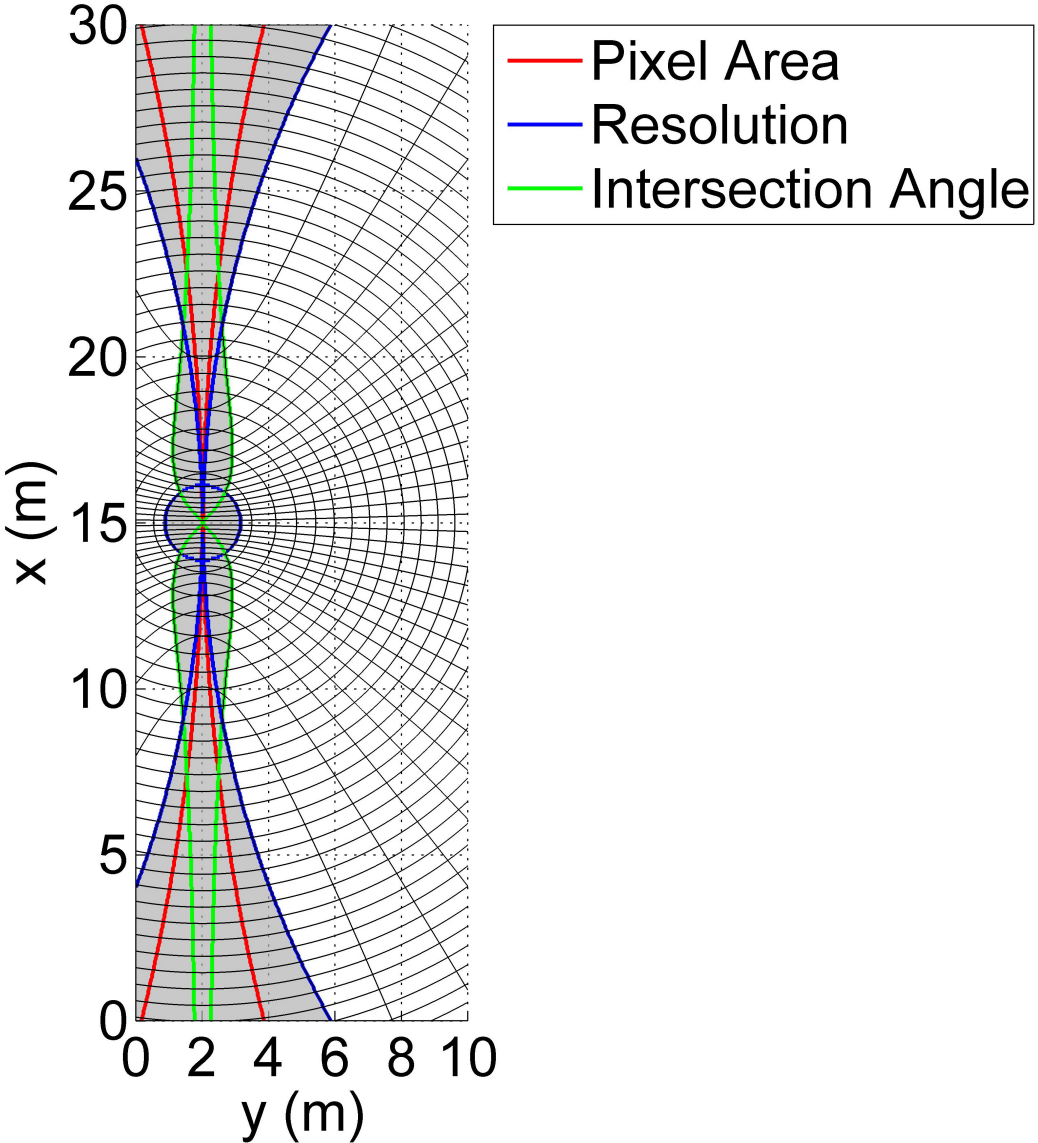
Plane OAED. Ambiguous area (shaded) and contributions: intersection angle (green contour), resolution (blue contour) and pixel size (red contour). For clarity, the distance between two close isolines does not represent the true system resolution.

**Figure 11.**
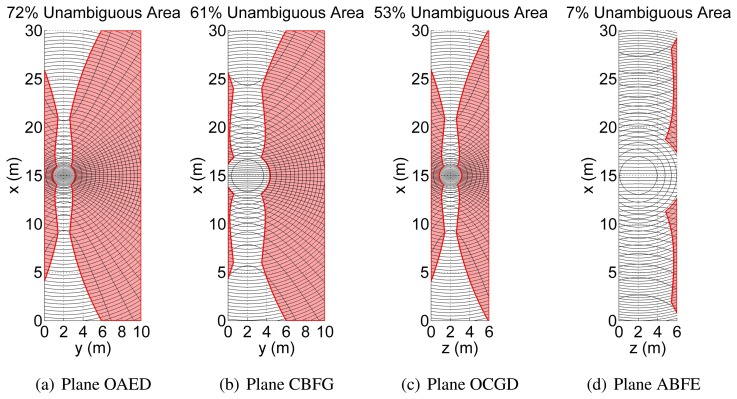
Total unambiguous area (in red, about 47% of the control volume surface) for the position and velocity reported in Table 7. Note that the observable walls are not depicted in the figure.

Quantitatively, a preliminary analysis of the mapping capability is carried out with the platform at a specific location. The antenna is located at position **P** with a velocity v (see Table 7) at half the integration time. The selected velocity and integration time give the theoretical azimuth resolution at a distance of about 3 m (and synthetic aperture equal to 0.5 m), but acceptable values are obtained even at longer distances, as shown in Figures 10 and 11. In more detail, Figure 10 shows the three terms that contribute to the ambiguous area (shaded) and the shape of the resolution element within the unambiguous area. The total unambiguous area is about 47% of the total area, and the walls having observable areas are depicted in Figure 11. It should be noted that points lying within areas, whose size depends on the distance (*i.e.*, the farther the wall, the larger the size), around the projection of the velocity direction on walls are not observable, owing to forward-looking ambiguities. In addition, points inside a circle, whose radius depends on the distance, around projections of the platform on the walls, are not observable, owing to the poor ground range resolution. Front and rear walls are not observable, as the vector normal to their surfaces is parallel to the velocity vector, thus resulting in parallel range and Doppler isolines. Furthermore, most of the wall ABFE is not observable. It is worth noting that even though the azimuth resolution satisfies the requirements of Table 6, the effects of both the ground range resolution and intersection angle Ωdue to the distance strongly affect the observation capability.

The presented results suggest that the whole control volume can be mapped by exploiting the platform agility to move and the point the beam.

### 4.2. Layover

Layover is a well-known geometric distortion of SAR images affecting targets that have the same range and velocity relative to the platform in three-dimensional space [40,45]. Layover does not affect the capability to image an area of interest, but can cause the inversion of the position of scatterers and geometric distortion, resulting in interpretation problems. With reference to the considered control volume, the most critical zones interested in layover are edges and angles generated by the intersection of two or three walls, which have at least two layover points [45]. However, this is not a specific problem of the proposed system, since it affects any radar observation, and SAR data processing algorithms do not typically remove layover areas. In addition, the exploitation of multi-aspect InSAR data has demonstrated good capabilities in terms of the recognition and removal of layover areas [47]. Even though these techniques have been tested on different scenarios, *i.e.*, layover generated by small and large buildings in urban areas, they are expected to be useful for the proposed system. Indeed, since it is expected that the required multi-aspect interferometric acquisitions will constitute the system operating mode in order to increase the percentage of the covered area within the control volume (see Section 4.1.1), the proposed and the successfully experienced techniques to cope with layover will be certainly exploited.

## 5. Conclusions

In this paper, the first steps towards the overall feasibility study and design of an innovative radar sensor for autonomous operations in GPS-denied indoor environments by flying small UAS have been taken. The work can be summarized as follows:

- After the state-of-the-art analysis of existing small SAR sensors, FMCW has been individuated as a suitable scheme to be exploited in combination with InSAR technology for applications requiring both high-resolution performance and compact and lightweight systems. Millimeter wavelengths have been selected thanks to their atmospheric penetration characteristics, even in environments with smoke and flames, and to limit antenna dimensions. The peculiar features of the FMCW scheme have been thus discussed, also giving a comparison with well-assessed pulsed SAR technology.
- Based on the FMCW features, a system design procedure has been achieved, outlining guidelines to trade-off the design choices based on the specific mission requirements and operative environments.
- Imaging peculiarities have been discussed in terms of the resolution.

The presented results demonstrate that high-resolution, high-quality observation of an assigned control volume is possible, provided that an adequate flight trajectory is selected. The advantage of FMCW with respect to the pulse architecture in terms of sampling frequency and real-time data handling suggests that the transmission of both raw data and processed images to the ground station could be easily achieved. It is clear that for autonomous navigation, onboard real-time data processing operations are required, such as interferogram formation, simultaneous localization and mapping procedures and structured data handling and storage, all of which are very demanding on the system processor. In addition, very long missions could produce an extremely large amount of data to be stored onboard. Nevertheless, it can be expected that future enhancements in miniaturization and customization of both processors and data storage devices will make the aforementioned problems affordable.

## Figures and Tables

**Table 1.** Basic design guidelines of the proposed innovative SAR system.

**Main Constraints**	

Mass	< 1 kg
Size	< 1500 cm^3^
Maximum dimension	< 30 cm
Antenna maximum length	< 10 cm
Power consumption	< 10 W
Real-time onboard processing	
**Expected Performances**	

3D Mapping without g	round truth
3D geometric resolution	10–20 cm
Field-of-view	Hemispherical
Operation in the presence of smoke and fire	
**Possible Technical Solutions**	

SAR	
Radar interferometry	
Millimeter wave radar	

**Table 2.** The main features of existing compact lightweight SAR systems (N/A = not available).

		**Mass (kg)**	**Size (cm^3^)**	**Power Consumption (W)**	**Transmitted Power (W)**	**Resolution (m)**	**Maximum Range (km)**	**Bandwidth (MHz)**	**Carrier Frequency (GHz)**	**Scheme**	**Onboard Real Time Data Processing**	**Single Pass Interferometry**
Lite-weight UAV Radar (LUAVR)	[19]	9	32,774	100	1	0.1	10	1800	35	FMCW SAR	Yes	No
MISAR	[11,20]	4	10,000	100	1	0.5	4	300	35	FMCW SAR	No	No
Brigham Young University (BYU) MicroSAR	[21,22]	2.7	2295.38	16	1	1	0.7	90	5.55	FMCW SAR	No	No
MiniSAR	[14]	14	250	250	60	0.3	10	3000	16.8	Pulsed SAR	Near-real time	No
NuSAR	[23,24]	8.62	N/A	160	25	0.3	0.7	500	9.75	Pulsed SAR	Yes	No
PicoSAR	[25,26]	10	10,797	300	1	0.3	20	768	9.7	Pulsed SAR	Yes	No
Radar de Apertura Sintética Miniaturizado Aéreo (MINISARA)	[27,28]	2.5	7296	N/A	1	0.07	2.97	2000	34	FMCW SAR	N/A	No
BYU MicroASAR	[29]	3.3	1880.71	35	1	0.75	N/A	200	5.43	FMCW SAR	No	No
SlimSAR	[30,31]	4.54	N/A	150	4	0.23	N/A	660	9.28	FMCW SAR	No	No
NanoSAR	[32]	0.91	1674	15	1	0.3	1	500	10.25	Pulsed SAR	No	No
NanoSAR B	[33]	1.59	1458.49	30	1	0.3	4	N/A	N/A	Pulsed SAR	No	Yes
NanoSAR C	[34]	1.18	1409.29	25	1	0.3	3	N/A	N/A	Pulsed SAR	No	Yes
Millimeterwave Radar using Analog and New Digital Approac (MIRANDA)	h [35]	2.2	4459.13	20	0.1	0.15	2	1000	94	FMCW SAR	No	No
ARBRES SAR	[15]	2.5	5950	50	N/A	1.5	N/A	100	9.65	FMCW SAR	N/A	Yes
MetaSensing SAR	[16]	N/A	N/A	N/A	N/A	0.4	N/A	450	9.65	FMCW SAR	N/A	Yes

**Table 3.** Input parameters for the system design.

**Symbol**	**Parameter**	**Unit**	**Minimum Value**	**Maximum Value**
*dr*	Range resolution	(cm)	10	20
*da*	Azimuth resolution	(cm)	10	20
*dh*	Height resolution	(cm)	10	20
*v*	Platform velocity	(m · s^−1^)	0.25	2.00
*θ*	Off-nadir angle	(°)	15	75
*θ_sq_*	Squint angle	(°)	−45	45
*R*_max_	Maximum distance	(m)	25.0	30.0
*R*_min_	Minimum distance	(m)	0.5	3.0
Δ*h*	Height difference between two points in adjacent range cells	(cm)	5	20
*N*_BIT_	Number of bits		16	32

**Table 4.** Constant parameters for the system design.

**Symbol**	**Parameter**	**Unit**	**Value**
*f_c_*	Carrier frequency	(GHz)	94
*λ*	Wavelength	(mm)	3.2
*c*	Speed of light	(m · s^−1^)	3 × 10^8^
*k_B_*	Boltzmann's constant	(J · K^−1^)	1.38 × 10^−23^
*T_N_*	Temperature of system	(K) (dB)	290
*F_N_*	Figure of noise		15
*SNR*	Signal-to-noise ratio	(dB)	20
*σ*^0^	Differential scattering coefficient	(dB)	−20
*η*	FMCW SAR duty cycle		1

**Table 5.** Selected working parameters.

**Symbol**	**Parameter**	**Unit**	**Value**
*dr*	Range resolution	(cm)	10
*da*	Azimuth resolution	(cm)	10
*v*	Platform velocity	(m · s^−1^)	0.50
*θ*	Off-nadir angle	(°)	60
*R*_max_	Maximum range	(m)	30
*R*_min_	Minimum range	(m)	1.5
*N*_BIT_	Number of bits		16
*dh*	Height resolution	(cm)	10
*B*	Transmitted bandwidth	(GHz)	1.50
***f****_S_*	Sampling frequency	(kHz)	68.327
*PRF*	Pulse repetition frequency	(Hz)	125
*d*	antenna width	(m)	0.01
*θ*_r_	antenna beamwidth in the range direction	(°)	18
*l_az_*	antenna length	(m)	0.02
*θ_az_*	antenna beamwidth in the azimuth direction	(°)	9
P*_T_*	Transmitted Power	(mW)	<1
*α*	Baseline roll angle	(°)	40
*B*_int_	Interferometric baseline	(cm)	3
Δ*φ*	Phase resolution at the interferometer	(°)	11
Δ*h*	Height difference between two points in adjacent range cells	(cm)	10

**Table 6.** Additional parameters for observation.

**Symbol**	**Parameter**	**Unit**	**Value**
*T*_int_	Integration time	(s)	4
Ω_min_	Lower bound on intersection angle	(°)	45
Ω_max_	Upper bound on intersection angle	(°)	135
A_pixel_	Pixel area threshold	(m^2^)	0.04
*k*_res_	Minimum required resolution	(m)	0.20

**Table 7.** Position and velocity of the antenna halfway through the integration time.

*P_x_* (m)	*P_y_* (m)	*P_z_* (m)	*v_x_* (m · s^−1^)	*v_y_* (m · s^−1^)	*v*_z_ (m · s^−1^)
15	2	2	0.5	0	0
